# The Role of MicroRNAs upon Epithelial-to-Mesenchymal Transition in Inflammatory Bowel Disease

**DOI:** 10.3390/cells8111461

**Published:** 2019-11-19

**Authors:** Éva Boros, István Nagy

**Affiliations:** 1 Institute of Biochemistry, Biological Research Centre, 6726 Szeged, Hungary; 2 Seqomics Biotechnology Ltd., 6782 Mórahalom, Hungary

**Keywords:** Inflammatory Bowel Disease (IBD), epithelial-to-mesenchymal transition (EMT), microRNAs

## Abstract

Increasing evidence suggest the significance of inflammation in the progression of cancer, for example the development of colorectal cancer in Inflammatory Bowel Disease (IBD) patients. Long-lasting inflammation in the gastrointestinal tract causes serious systemic complications and breaks the homeostasis of the intestine, where the altered expression of regulatory genes and miRNAs trigger malignant transformations. Several steps lead from acute inflammation to malignancies: epithelial-to-mesenchymal transition (EMT) and inhibitory microRNAs (miRNAs) are known factors during multistage carcinogenesis and IBD pathogenesis. In this review, we outline the interactions between EMT components and miRNAs that may affect cancer development during IBD.

## 1. Introduction

Inflammatory Bowel Disease (IBD) is a group of multifactorial disorders characterized by chronic inflammation along the digestive tract [1]. Two main types of IBD are Crohn’s disease (CD) and ulcerative colitis (UC), whose common symptoms—such as bloody diarrhea, abdominal pain, malabsorption and fatigue—significantly reduce the quality of life [2]. The main difference between CD and UC is the location of the lesions in the gastrointestinal tract. In CD patients, inflammation can manifest anywhere from the mouth to the anus, while in UC it is limited to the colon [2]. The exact pathogenesis of IBD is still incompletely characterized; presumably, it is the outcome of the complex interference between genetic, microbial, environmental, and lifestyle factors [3]. Genome-wide association studies (GWAS) often identify novel genetic susceptibility loci for IBD: Up to now, more than 240 such loci have been reported [4,5,6]; however, part of these risk factors considerably alter between trans-ancestry populations [7,8]. Importantly, however, the presence of IBD susceptibility mutations is insufficient to break the homeostasis of the gut, as twin studies showed low concordance between these factors and manifestation of the disease [9]. The main relevance of GWAS is that genetic factors have an effect on those genes that are responsible for the interaction between host and environment (e.g., NOD2 or FUT2) [9]. In addition, the importance of the microbiome and lifestyle is indisputable in the pathogenesis of IBD, but these circumstance cause disease merely in genetically susceptible persons [9]. Over the intestinal and systemic complications [10,11], long-lasting inflammation increases the risk of colorectal cancer (CRC), and these malignancies account for 10 to 15% of deaths in IBD patients [12]. Prolonged inflammation contributes to tumorigenesis in multiple manners [13]: proinflammatory genes and pathways promote cancer niche formation [14], while regulatory microRNAs enhance the imbalance [15]. During the repair of damaged tissues, epithelial-to-mesenchymal transition (EMT) plays a role in wound healing; however, EMT activation is also involved in cancer development and may promote the progression of IBD-related CRC [16]. In the context of cancer, EMT plays a role in the formation of invasive mesenchymal-like cells and facilitate phenotypic plasticity that is required for the development of cancer stem cells (CSCs) [17]. Importantly, the expression of genes related to EMT is regulated, among others, by miRNAs, which may also affect EMT function in tumor progression [18].

## 2. Risk Factors of CRC in IBD

Colorectal cancer is the third most commonly diagnosed cancer type, with the highest age-standardized incidence rate in Hungarian population, notably 51.2 cases per 100,000 persons per year [19]. Chronic inflammation of the colon drives the formation of colitis-associated colorectal cancer (CAC) that has higher malignance rate than sporadic CRC [20]. Incidence of colorectal cancer in IBD is altered accordingly to the disease duration that proved to be an important risk factor [21]. Based on a retrospective cohort study, which was published in 2019, the cumulative risk of CRC was 0.3% at 10 years, 1.3% at 20 years, and 5.9% at 30 years after the onset of UC [22]. Previous studies differ in the exact incidence rate, and older ones estimated higher values than recent reports [21,23], but they all underline a positive correlation between IBD and CRC [16,24,25]. Not surprisingly, the prevalence of colorectal cancer alters between UC and Crohn’s disease (CD), since the localization of the lesion(s) differ along the gastrointestinal tract, yet colonic CD also increases the risk of CRC [12,26].

**Figure 1:**
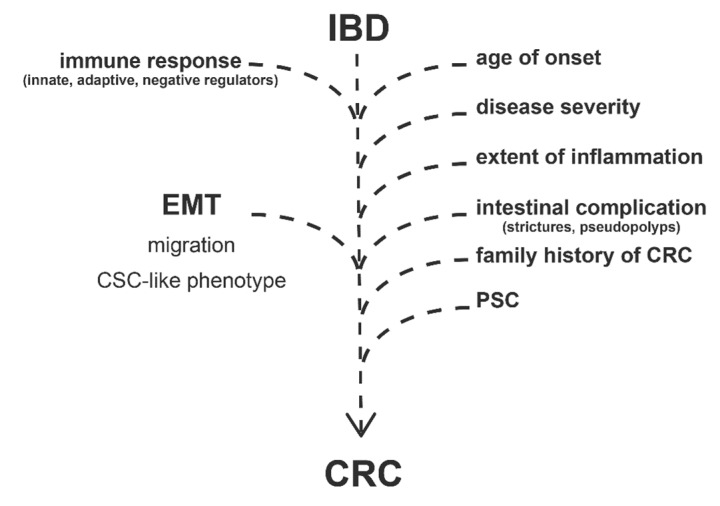
Risk factors and molecular processes contributing to the development of colorectal cancer in Inflammatory Bowel Disease (IBD) patients. EMT = epithelial-to-mesenchymal transition; CRC = colorectal cancer; PSC = Primary Sclerosing Cholangitis; CSC = cancer stem cells.

Apart of the disease duration, development of CRC in IBD patients depends on many other clinical factors (Figure 1). Previous reviews extensively discussed the following risk factors: age of onset, disease severity, extent of inflammation, presence of intestinal complications (strictures, pseudopolyps), family history of colorectal cancer, and PSC (Primary Sclerosing Cholangitis) [12,16,24,25].

Based on a cohort of 9505 individuals with IBD, IBD per se increases the risk of CRC 3- to 5-fold, while family history of CRC in the first degree relatives causes an additional 8-fold elevation [27]. Furthermore, Ryan et al. examined the genetic correlation between IBD susceptibility SNPs (single-nucleotide polymorphisms) and formation of colon cancer and identified STAT3 locus as a potential link [28].

In addition to the clinical risk factors, the development of CRC is highly related to signaling pathways, regulatory mechanisms, and gene expression alterations which are otherwise characterizing IBD. Most importantly, the activation of innate and adaptive immune response (Figure 1) by the induction of pattern recognition receptors (e.g., TLRs and NLRs) or increased expression of cytokines and chemokines eventuates infiltration of immune cells and inflammatory response [24,29,30].

Negative regulators of immune response have been also revealed as potential participants in the IBD to CRC progression (Figure 1) [24]. In our previous study, we observed the altered expression of TAM receptors (TYRO3, AXL, and MERTK) in IBD, which are known negative regulators of inflammation and associate with cancer progression [31,32]. In the case of tumorous malignancies, AXL expression negatively correlates with lifespan, and its elevated level is the predictor of a bad clinical outcome [33,34]. AXL plays a role in the regulation of NF-κB and JAK-STAT pathways, thereby regulating inflammation; moreover, it is able to activate SNAI1/2, ZEB2, and TWIST transcription factors, thus inducing epithelial-to-mesenchymal transition; furthermore, by the induction of MMP9, AXL increases cell motility and invasiveness [35,36]. Both mRNA and protein levels of AXL significantly increased in the inflamed colon regions of rat experimental colitis (2,4,6-trinitrobenzene sulfonic acid (TNBS) induced rat model of colitis); in addition, elevated AXL gene expression was detected in colonic lesions of IBD patients [37]. At the site of inflammation, pleiotropic AXL may have a role as a negative regulator of innate immunity, as well as phagocytic receptor in the reconstruction of tissue homeostasis [38]. As an inducer of epithelial-to-mesenchymal transition, AXL may also enhance the risk of colorectal cancer in IBD patients [36].

In summary, disease duration, clinical risk factors, inflammatory signaling, and EMT pathways cooperatively facilitate CRC progression in IBD patients (Figure 1). In this review, we focus on EMT-related genes and their transcriptional regulators (such as microRNAs) that possibly affect IBD to CRC progression, with special attention given to the interplay of inflammation and EMT upon tumorigenesis.

## 3. Inflammation, EMT, and Tumorigenesis

Cancer progression is mostly triggered by somatic mutations that are a consequence of environmental effects. In many cases, chronic infection, UV radiation, obesity, extreme diet, airway pollutants, smoking, or autoimmune diseases provoke carcinogenesis. Their common feature is that they bring on chronic inflammation, and this abnormal defensive mechanism destroys tissue homeostasis [39].

In 1863, Rudolf Virchow described the association between chronic inflammation and tumorigenesis, while he observed the enhanced infiltration of immune cells into the cancer microenvironment [40]. In the 1990s, numerous studies proved the importance of immune cells that are regulating inflammation, cytokines, chemokines, and growth factors in the development of cancer [41]. Moreover, widely used IBD therapeutics that aim to control inflammation have a beneficial side effect. Namely, these anti-inflammatory drugs, such as 5-ASA (5-Aminosalicylate) or thiopurines, reduce the risk of IBD-related CRC [42,43], which is additional evidence to support the correlation between inflammation and tumorigenesis.

Tumorigenesis is a complex process that usually initiates from a single cell which, after several divisions, avoids differentiation and apoptosis, finally creating a cell mass that accumulates novel mutations. At the initiation stage, lack of blood supply causes necrosis, and cell debris induces tissue repair and angiogenesis that helps the evolution of supporting circumstances for tumor formation [44]. Creation of the tumor microenvironment (TME) is essential for tumorigenesis, that is the result of cooperation between effector molecules (cytokines, chemokines, transcription factors) and different cell types [14]. Cellular components of TME formation are principally immune cells, among them tumor-associated macrophages, dendritic cells, and myeloid cells; in addition, fibroblasts, stromal, and endothelial cells are also essential [13].

In the inflamed tissue, infiltrated immune cells secrete cytokines and chemokines that activate several signaling pathways through the induction of transcription factor(s), such as NF-κB. The canonical NF-κB pathway is mostly activated by TNFα, IL1α/β, and TLR ligands and plays a role in the regulation of inflammation, cell division, epithelial-to-mesenchymal transition, angiogenesis, and metastasis related genes [13]. By regulating extracellular matrix (ECM) rearrangement, matrix metalloproteinases (MMPs) are crucial factors in TME formation: it is known that the expression of MMPs is induced during inflammatory response, as well as EMT [45]. Hence, the significance of inflammatory pathways and molecules is well known in carcinogenesis and TME formation, as well as in the concept of cancer stem cells (CSCs) [14,20,44]. According to the CSC concept, a minor population of cancer cells have the unique ability to seed new tumors, as well as renew and differentiate like stem cells [20,46]. This phenomenon was also observed in CRC; for example, IBD-related induced signaling, such as the WNT-β-catenin, TNFα-NFκB, and IL6- STAT3 pathways, stimulate CSCs [20,47]. Genome sequencing uncovered numerous genetic changes underlying phenotypic plasticity of CSCs, yet this process may also be undergone without mutations, which points toward the existence of epigenetic regulatory mechanism(s) [46]. Phenotypic alteration between tumor forming CSCs and bulk tumor cells or non-CSCs (NCSCs) may also be regulated by epithelial-to-mesenchymal transition [46]: induction of EMT by transcription factors or TGFβ raise stem-like properties in epithelial tissues [17]. CSC phenotype was also observed in EMT-induced tumor cells, such as nontumorigenic mammary epithelial cells [48,49,50]. The overexpression of EMT-inducing transcription factor SNAI1 increased CSC-like phenotype in human CRC cells [48], while in immortalized human mammary epithelial cells, EMT promoted CSC generation [49]. These data clearly establish a link between EMT and the acquisition of stem-like features, yet the molecular mechanism(s) underlying remains largely unexplored.

Milestones of IBD-associated CRC are low-grade dysplasia (LGD) followed by high-grade dysplasia (HGD) that finally develop into adenocarcinoma [12,16]. From a clinical perspective, the most critical aspect is the formation of invasive cancerous cells that are capable to break through the basal lamina, wherein EMT is a key process [44]. Timing of metastasis formation is unknown, but according to a recent report, early disseminated cells seed metastasis before the carcinoma is detectable (smaller than 0.01 cm^3^) [51]. In a mouse model of pancreatic cancer, pancreatic cells with mesenchymal and stem cell characteristics were observed in the bloodstream and seeded the liver before the presence of detectable histological signs of malignance in the pancreas. Moreover, EMT was activated during inflammation that enhanced the number of circulating pancreatic cells [52].

Taking together, EMT is related to different aspects of tumor progression, from supporting TME formation through phenotypic alteration of CSCs till triggering metastasis. Hence, the examination of EMT related mechanism(s) is important at every stage of inflammation and cancer progression.

## 4. Molecular Mechanism of Epithelial-to-Mesenchymal Transition

**Figure 2:**
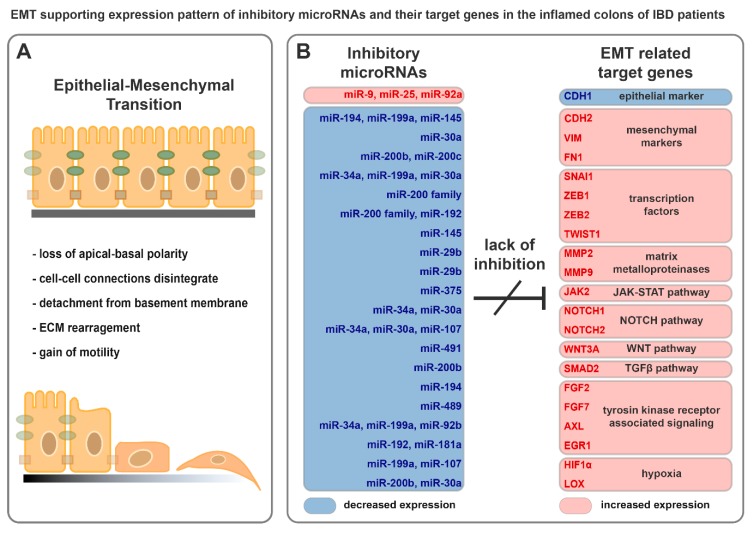
Downregulation of inhibitory microRNAs support epithelial cell transition into mesenchymal-like phenotype. (**A**) Schematic representation of EMT progression. (**B**) Expression pattern of inhibitory microRNAs and their possible EMT related target genes in the inflamed colons of IBD patients.

During EMT, epithelial cells lose their apical-basal polarity, cell-cell connections disintegrate, and instead of epithelial markers, they express mesenchymal constituents (Figure 2A). Detachment of epithelial cells from the underlying basement membrane results in motility, while matrix metalloproteinases degrade extracellular matrix components. The emergent invasive mesenchymal cells are resistant to senescence and apoptosis [53]. Under physiological conditions, EMT is indispensable for embryogenesis and tissue regeneration but also plays a role in the development of tumorigenesis and metastasis formation [54].

### 4.1. Disengagement from the Bondage of Junctions

Breakdown of cell–cell connections is the consequence of the reduced expression of claudins (CLDN3, -4, and -7) and occludin (OCLN), as well as the degradation of E-cadherin (CDH1), in the membranes [55]. Furthermore, the reduced expression of polarity complex proteins, such as CRB3 and LGL2, causes loss of polarity [54]. In contrast, newly evolved mesenchymal cells express N-cadherin (CDH2), vimentin (VIM), and fibronectin (FN1), providing invasiveness [17]. During this process, the appearance of cells changes from cobblestone to spindle-shape (Figure 2A) [17].

### 4.2. Control of EMT by Transcription Factors

Decreased expression of epithelial- and induction of mesenchymal markers is regulated by SNAI (snail family transcriptional repressor, SNAI1/2), TWIST (twist family bHLH transcription factor 1, TWIST1), and ZEB (zinc finger E-box binding homeobox, ZEB1/2) transcription factors, often referred to as EMT transcription factors (EMT-TFs) [17,56]. EMT-TFs shift the transition to mesenchymal stage by the repression of CDH1 and the regulation of other EMT related genes, such as matrix metalloproteinases (e.g., MMP2, MMP9, MMP14, or MMP15), which, in turn, promote EMT by the rearrangement of extracellular matrix components, thereby increasing cell motility [54,56,57].

### 4.3. Influence of Signaling Pathways

EMT is associated with multiple inflammation related signaling pathways, such as JAK-STAT, NOTCH, or WNT pathways, furthermore it is linked to the TGFβ-induced MAPK cascade and plays a role in the regulation of growth factors through receptor tyrosine kinases [17,54].

The importance of the JAK-STAT signaling pathway has been proved in many pathological conditions and diseases, from immune deficiencies to cancer. This pathway is a signal transducer of cytokine-, interferon-, and growth factor transmembrane receptors, related to diversified function [58]. From the aspect of EMT, elevated expression of JAK2 supports the transition to mesenchymal stage [59].

NOTCH family members (NOTCH1, -2, -3, and -4) are transmembrane receptors taking part in regulation of proliferation, differentiation, and apoptosis [60]: all of them were confirmed as regulators of EMT. NOTCH1 enhance EMT by the activation of SNAI2 [61], while the knockdown of NOTCH2 leads to increased E-cadherin and decreased SNAI1 and VIM levels [62]. In addition, NOTCH4 inhibits EMT through transcription repressor of HEY1 that is a positive regulator of tumor suppressor p53 [63,64]. Interestingly, the effect of NOTCH3 seems to be reverse as an inhibitor of EMT by the activation of Hippo/YAP pathway [65].

The WNT pathway induces EMT by the transcriptional activation of SNAI2 and TWIST1 transcription factors [66] and, furthermore, by the activation of oncogenic IQGAP1 that promotes cell proliferation [67]. By WNT3A ligand binding, the WNT receptor is involved in the upregulation of N-cadherin and downregulation of E-cadherin [68].

The effect of TGFβ on EMT was observed in cell cultures, where TGFβ-treatment caused overexpression of mesenchymal genes and induced transformation of epithelial cells to elongated mesenchymal cells [69]. TGFβ-activated EMT occurs in alternative manners, such as the activation of SMAD, Ras/MAP kinase, Rho-like GTPases, or PI3 kinase/Akt signaling pathways [70]. According to Pang et al., after the TGFβ1 induced EMT, the newly-formed mesenchymal cells become capable for migration through the lymphatic vessels by the contribution of CCR7 and CCL21, which ensure the transmigration through the endothelial cells [71].

Tyrosine kinase receptors facilitate the effect of growth factors on EMT [54]. Similarly to TGFβ, they can activate several downstream signal transducers, thereby connecting to the PI3K-AKT, ERK/MAPK, p38/MAPK, and JNK pathways [54].

Fibroblast growth factor receptors (FGFRs) belong to transmembrane receptor tyrosine kinases; FGF signaling implicates the regulation of cell differentiation, survival, tissue regeneration, and also EMT [72]. Fibroblast growth factor 1 (FGF1) directs the transition to mesenchymal stage by the destabilization of desmosomes and induction of the expression of integrins and MMP13 [54]. Through FGFR/ERK signaling, FGF2 induces EMT and tumor growth in ESCC [73]. FGFR2 activation by FGF7 drives epithelial-to-mesenchymal transition in human keratinocytes, as well as head and neck cancer cells [74,75,76].

AXL, a member of TAM tyrosine kinase receptors, has been shown to act via PI3K, MAPK, and PKC pathways and is able to activate NF-κB and JAK/STAT signal transductions [38,77]. AXL expression correlates with mesenchymal phenotype, and its knockdown mitigates SNAI2, TWIST1, and ZEB1 expression, while enhancing the E-cadherin level [35].

Early growth response 1 (EGR1) transcription factor is connected to different signal transducer cascades, such as ERK/MAPK or MET/MAPK pathways, and responds to broad range of stimuli, e.g., growth factors, reactive oxygen species (ROS), or oxygen deprivation, hence play a role in cell proliferation, differentiation, apoptosis, and cancer progression as a multifunctional switch [78,79]. EGR1 is able to activate the promoter of SNAI1; furthermore, EGR1 and SNAI1 collaborate upon induction of MMP9 and ZEB1 expression [54,80].

Limited oxygen supply of inflamed tissues and TME leads to elevated HIF1α production that promotes EMT through the NOTCH pathway and by the regulation of AXL and TWIST [54,60,81,82]. Hypoxic conditions enhance EMT and the expression of lysyl oxidase (LOX) that modulates β1 integrin signaling. Furthermore, knockdown of LOX increased E-cadherin and decreased vimentin expression in gastric cancer [83].

### 4.4. Role of EMT in IBD

IBD is characterized by long-lasting inflammation of the gastrointestinal track that fosters fluctuation of tissue injury and healing. As a result, intense rearrangement of ECM promote the formation of intestinal fibrosis [84]. Significance of EMT was examined mainly in the aspect of fibrosis and fistulae formation in IBD patients [84,85,86]. Fistulae of CD patients are partly constructed from mesenchymal-like transitional cells (TCs), which have an EMT-inducing gene expression pattern that seems to promote fistulae formation [86]. Immunohistochemical staining revealed high protein expression of SNAIL, SLUG, and FGF2 in IBD fistulae [85]. Presence of CD-68 positive mononuclear cells imply the inflamed status of the fibrotic tissues of CD patients, where the expression of TGFβ1 and SLUG was significantly elevated [87].

Increased expression of EMT activating protein coding genes was reported both in the inflamed colon samples of rat experimental colitis as well as IBD patients [37,88]. For example, expression of growth factors (FGF2 and FGF7), signal transducers (EGR1, NOTCH2, JAK2, and HIF1α), EMT inducing transcription factors (ZEB2 and SNAI1), the extracellular matrix remodeler MMP9 and mesenchymal markers (VIM and LOX) were highly elevated in the inflamed colon tissues; in contrast, decreased expression of the epithelial marker CDH1 was observed (Figure 2B) [37,88].

## 5. MicroRNAs

In the last two decades non-coding microRNAs came into focus of cancer research since their altered expression was reported in numerous tumor types including colorectal cancer [89]. miRNAs play a role in the posttranscriptional regulation of their target mRNA(s) and, so, indirectly take a part in cell division, cell differentiation, and cell fate processes [90].

### 5.1. Biogenesis and Function of MicroRNAs

miRNAs are short, 18–21 nt long, evolutionary-conserved RNA molecules. The primary transcripts of miRNAs (pri-miRNAs) are usually a few thousand nucleotide-long transcripts that are transcribed from coding or intergenic regions of the genome. During their maturation, intermediates translocate from the nucleus to the cytosol and are cleaved by several enzymes until miRNAs achieve the mature functional form [91]. Mature miRNAs hybridize to the 3′ UTR (untranslated region) of the target mRNAs and execute posttranscriptional modifications: In the case of exact match, they initiate mRNA degradation, while imperfect complementarity results in incomplete translation [92].

Seed regions of miRNAs are responsible for target recognition: this is a strongly conserved 6 nt long sequence from the 2nd to the 7th nucleotide position of the given miRNA. Based on the similar seed sequences, miRNAs are catalogued into miRNA families. As a result of the same seed region, members of a given miRNA family play a role in the regulation of nearly the same mRNA target pool. Numerous miRNAs form clusters in the genome and are transcribed as one common pri-miRNA; hence, their transcription is cooperatively regulated. Importantly, the effect of miRNAs is also redundant: A single mRNA can be regulated by several different miRNAs; in addition, a given miRNA is able to inhibit numerous mRNAs. Notably, regulation by miRNAs does not operate as a switch; instead it works like a fine-tuner of gene expression [93].

### 5.2. Role of miRNAs in IBD

Understanding the molecular background of IBD pathogenesis faces serious difficulty because of the multifactorial characteristic of the disease. Genetic, microbial, and/or environmental risk factor(s), individually, are inadequate to support the pathogenesis of CD or UC. The pleiotropic effect of miRNAs by their wide palette of target mRNAs may empower microRNAs to interconnect these seemingly independent factors that eventuate the flare up of IBD.

Specific miRNA signatures were observed in IBD associating with canonical signaling pathways regulating autophagy, inflammation, fibrosis, or EMT [94]. For example, the NOD2 receptor—the first identified CD risk gene [95,96]—has a crucial function in the regulation of autophagy is a direct target of miR-192; in addition, the IBD associated rs3135500 SNP affects the binding site of miR-192 in the 3′ UTR of NOD2 [97]. Upon regulation of inflammatory response, miR-155 inhibits the negative regulator of JAK/STAT signaling SOCS1, hence elevating expression of miR-155 in the involved tissues of IBD patients enhances inflammation [94]. Because of their potential anti-inflammatory effects, miRNAs that target innate and adaptive immune response-associated genes are intensely studied with respect to IBD [98]. Conversely, increased risk of CRC in IBD patients justifies the significance of the research on the fibrosis-, EMT-, and cancer-related miRNAs [94]. Currently, the most widely studied EMT regulating group of microRNAs are the members of the miR-200 family, where altered expression was described in the inflamed mucosa of IBD patients [99].

### 5.3. miRNAs Having Target mRNAs Related to EMT with Potential Role in IBD Pathogenesis

Here, we have collected those miRNAs and their target mRNAs that are related to EMT and may have potential role in the progression in IBD. Even though the regulatory role of a given miRNA on its target gene(s) may have been validated in different model systems (Table 1 and references within), the expression pattern of miRNA-mRNA target pairs were reported in the inflamed colon samples of IBD patients (Figure 2B).

***CDH1/E-cadherin*** Decreased expression of E-cadherin is crucial upon transition to mesenchymal stage. Known posttranscriptional inhibitors of CDH1 are miR-9, miR-25, and miR-92a. Expression of miR-9 is upregulated by oncogenic transcription factors MYC and MYCN, leading to increased motility of breast cancer cells. Besides the repression of CDH1 through β-catenin pathway, the elevated level of vascular endothelial growth factor (VEGF) induces angiogenesis [100]. miR-25 and miR-92a belong to the same, namely miR-92 microRNA, family and are highly conserved during evolution. miR-92 family is one of the firstly discovered oncogenic miRNA family; its aberrant expression is reported in many cancer types; for instance, it is found in colon tumors [101]. CDH1 is a direct target of both miR-25 and miR-92a, in which levels were highly increased in carcinoma cells, where the expression of E-cadherin was repressed, leading to the increased invasiveness of cells [102,103]. Elevated expression of miR-9 in inflamed colon tissue and higher level of miR-92a in stool samples from IBD patients has been reported that may cause reduction of CDH1 and induce EMT in IBD [37,104,105].

***CDH2/N-cadherin*** Characteristic biomarker of the mesenchymal stage in EMT is N-cadherin that is overexpressed in Crohn’s strictures [106]. miR-194 is a direct inhibitor of CDH2, and its repressed expression promotes motility of the mesenchymal-like cancer cells [107]. Expression level of miR-194 is significantly decreased in colonic tissue of both UC and CD patients [108]. Moreover, miR-199a and miR-145, additional regulators of CDH2, were also repressed in the inflamed colons of IBD patients [37,109,110,111].

**Figure 3:**
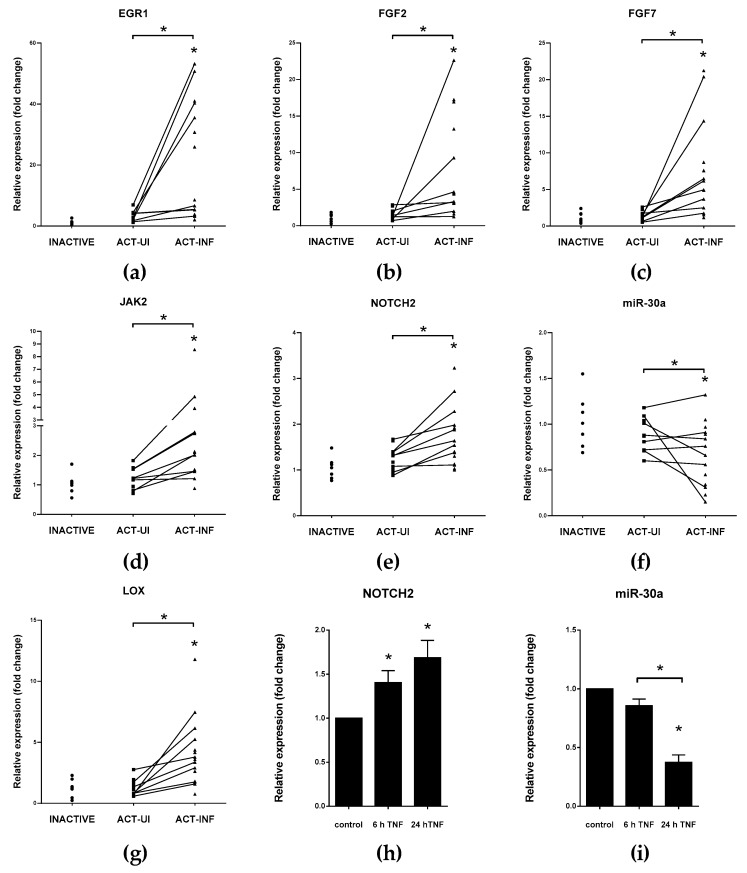
Distinct expression of genes and microRNA miR-30a regulating epithelial-to-mesenchymal transition (EMT) in IBD patients (**a**–**g**) and TNFα-triggered HT-29 cells (**h**,**i**). The relative expression of genes involved in epithelial-to-mesenchymal transition EGR1 (**a**), FGF2 (**b**), FGF7 (**c**), JAK2 (**d**), NOTCH2 (**e**), and LOX (**g**), as well as microRNA miR-30a (**f**) is shown from inactive (left, n = 7), active uninflamed (ACT-UI, middle, n = 12), and active inflamed (ACT-INF, right, n = 15) colon samples of IBD patients. The relative expression of NOTCH2 (**h**) and miR-30a (**i**) in TNFα-triggered HT-29 cells after different incubation times. For a detailed description of materials and methods, please see [37]; * *p* < 0.05.

***VIM/vimentin*** Mesenchymal marker vimentin is highly upregulated in inflamed colonic mucosa of IBD patients which negatively correlates with the decreased expression of miR-30a (Figure 3f and [37]). In gastric cancer cells, the miR-30a based inhibition of VIM is induced by tumor suppressor RUNX3 transcription factor, in addition, decreased miR-30a level enhance invasion ability of cells [112]. Notably, miR-30a is also repressed by TNFα in HT-29 human colon cancer cell line (Figure 3i).

***FN1/fibronectin*** Primary component of mesenchymal cells is the ECM protein fibronectin [113]. miR-200b and miR-200c play a role in the posttranscriptional regulation of FN1 by direct binding to its 3′ UTR region; hence, their downregulation triggers EMT [114,115]. Reciprocal expression of FN1 and miR200b/c was observed in the inflamed colon of IBD patients [116,117].

***SNAI1/snail*** EMT-related transcription factor, snail, is a key regulator of the transition to mesenchymal stage. Known posttranscriptional regulators of SNAI1 are members of the miR-34 family, which have critical function in the regulation of cell cycle, formation of metastasis and resistance against chemotherapy [143]. Expression of miR-34a is regulated by p53 transcription factor, and both of them induce apoptosis [128]. A negative feedback loop exists between miR-34 and SNAI1: ectopic overexpression or p53 induces elevation of miR-34 that, in turn, downregulates SNAI1; in contrast, SNAI1 inhibits miR-34 transcription by the repression of its promoter [118]. Strongly conserved miR-199a has the same seed region as miR-34a. Upon inhibition of CDH2, miR-199a also suppresses the expression of SNAI1 [109]. Furthermore, miR-30a, a known regulator of vimentin, is able to bind to the 3′ UTR of SNAI1 [119,120]. Expression of miR-34a, -199a, and -30a decreased, and mRNA level of SNAI1 significantly increased in the inflamed colons of IBD patients ([37] and Figure 3f).

***ZEB1 and ZEB2*** The role of MIR-200 family members (miR-200a/b/c,-141,-429) in the regulation of EMT is extensively studied: it is known that these miRNAs delay EMT by the inhibition of ZEB1 and ZEB2 transcription factors [121]. In contrast, ZEB2 inhibits the transcription of miR-200b generating a negative feedback loop. ZEB2 is also regulated by miR-192 that is repressed by EMT activating TWIST1 transcription factor [18,122]. Elevated expression of ZEB2 along with decreased levels of miR-192 and miR-200b in the inflamed regions of rat experimental colitis and colons of IBD patients were reported [37,88].

***TWIST1*** Overexpression of TWIST1 has been observed in many cancer types and it plays a role in tumor initiation and EMT [144]. By the binding to the 3′ UTR, miR-145a, -151, and -337 are able to downregulate TWIST1 [123]. T-helper cells derived from inflamed colon tissues of CD and UC patients express high amount of TWIST1 that contributes to the regulation of cytokine expression [145]. Expression of tumor suppressor miR-145 decreased in the inflamed colon of UC patients [110]. In addition, reduced expression of another tumor suppressor microRNA miR-145 has been described in lung, pancreatic, prostate, ovarian, breast, and colorectal cancers [143].

***MMPs/matrix metalloproteinases*** Upon EMT, MMPs play a role in the rearrangement of ECM. Anti-fibrotic effect and reduced expression of miR-29b has been reported in CD patients [146]; furthermore, the loss of miR-29b promotes mesenchymal phenotype by the insufficient inhibition of MMP2 and MMP9 in colon and breast cancer cells, respectively [124,125]. Enhanced expression of MMP9 is characteristic in the inflamed colon tissues of both rat experimental colitis and IBD patients [37,88].

***JAK2/Janus kinase 2*** EMT-inducing transcription factor SNAI1 reduces the transcription of miR-375 thereby boosts the expression of key participants of inflammatory response, such as JAK2, MAP3K8, and TP53 [126,127,147,148]. ZEB1 takes part in the transcriptional regulation of miR-375, and the level of this microRNA negatively correlates with EMT in prostate cancer cells [149]. Expression of miR-375 was significantly reduced in inflamed tissues of IBD patients compared to healthy controls or intact colon tissues of IBD patients and in rat experimental colitis, while the mRNA level of its target JAK2 significantly increased ([88,150] and Figure 3d).

***NOTCH family*** Tumor suppressor effect of miR-34 manifests through the expression of its target mRNAs, for instance by the direct inhibition of EMT inducer NOTCH1/2 or proto-oncogene transcription factor C-MYC [128]. Ortega et al. reported a potential regulatory loop connecting the oncogenic signaling of MYC and NOTCH to each other through posttranscriptional regulation by miR-30a [129]. MYC suppresses the transcription of miR-30a, thereby releasing microRNA-induced repression of NOTCH1 and NOTCH2 [129]. Another suppressor of NOTCH2 is miR-107 that is under the control of TP53, accordingly, inverse miR-107—NOTCH2 expression plays a role in cell growth and proliferation [130,151]. In the inflamed colon tissues of IBD patients, rat experimental colitis, or TNFα triggered HT-29 colonic epithelial cells, the same reciprocal expression pattern was observed: increased NOTCH2 (Figure 3e,h) level was accompanied by downregulation of miR-30a (Figure 3f,i), miR-34,and miR-107 [37,88].

***WNT3A/Wnt family member 3A*** Through the inhibition of WNT3A, miR-491 plays a role in the regulation of Wnt3a/β catenin signaling in gastric cancer, with its activation observed in the early phase of colitis-associated tumor development [131,152]. Besides, miR-491 regulates SNAI1 and metastasis in gastric cancer cells [153]. Elevated expression of WNT3A and mitigation of miR-491 may support EMT in IBD [154,155].

***SMAD2/SMAD family member 2*** Key components of TGFβ-induced EMT is SMAD2 that is under the control of miR-200b in intestinal epithelial cells. Additionally, depletion of SMAD2 by overexpression of miR-200b caused the downregulation of vimentin. Reciprocal correlation between TGFβ signaling/TGFβ expression and miR-200b level was observed in IBD that may also promote EMT [99].

***FGF signaling*** Through the activation of ERK and MAPK signal transduction pathways, after the TGFβ induced isoform switching of fibroblast growth factors receptors (FGFRs), FGF2 induces the rearrangement of ECM by the activation of microenvironment proteases, hence supporting EMT [17,156,157]. While FGF2 is also a direct target of miR-194 [132], FGF7 is under the posttranscriptional control of miR-489 [133]. In the inflamed colon sections of rat experimental colitis and in IBD patients, elevated expression of FGF2 and FGF7 were observed ([88] and Figure 3b,c). In contrast, the expression of both miR-194 and miR-489 decreased in mucosal tissue of UC patients that may contribute to the EMT-promoting effect of FGF signaling [108,158].

***AXL*** Several reports prove the role of tyrosine kinase receptors in the process of EMT, including the significance of AXL, a member of TAM family [17,33,54]. Both miR-199a and miR-34a regulate the expression of oncogenic AXL [134,135]; in contrast, tumor suppressor PPARγ induced miR-92b reduced AXL expression in fibroblasts [136,159]. Notably, AXL has an inverse expression pattern with its inhibitory microRNAs miR-34a and miR-199a in the inflamed colons of IBD patients [37].

***EGR1/early growth response 1*** Molecular components of inflammation and EMT overlap and collaborate with each other. EMT activating transcription factor TWIST1 inhibits mir-192 transcription, thereby inducing the expression of its target EGR1, which, in turn, enhances the level of proinflammatory cytokines, chemokines, and growth factors, such as IL-6, CXCL8, CXCL1, and FGF2 [122]. Subsequently, IL-6 intensifies inflammation by triggering the decrease of miR-200 family member miR-200c [160]. Another known inhibitor of EGR1 is miR-181a that additionally plays a role in the regulation of TNFα, which is a general target in IBD therapy [137,161,162]. Expression of EGR1 significantly increased in the inflamed colons of IBD patients (Figure 3a) and rat experimental colitis, where miR-192 level also decreased [37,88]. Interestingly, the expression of miR-181a changed accordingly to the phase of progression from non-neoplastic to dysplasia or from dysplasia to cancer in CD patients, with robust reduction of miR-181a level supporting CRC development [163].

***HIF1α/hypoxia inducible factor 1 alpha*** Oxygen deprivation of damaged tissues induces the expression of HIF1α that regulates SNAI1, ZEB1, and β-catenin, thereby activating EMT and contributing to fibrosis formation and colorectal cancer development [164,165]. Beside the aforementioned CDH2, SNAI1, and AXL, miR-199a also inhibits HIF1α [138,139]. An additional regulator of HIF1α is miR-107 [140]. Elevated HIF1α level enhances inflammation and decreases barrier integrity of the involved intestinal tissues in IBD [37,166].

***LOX/lysyl oxidase*** Hypoxic conditions lead to the elevated expression of LOX that play a role in the remodeling of ECM components and are associated with fibrosis in IBD [83,167]. miR-200b and miR-30a are validated regulators of LOX, and both of them are able to decrease invasiveness of tumor cells by the inhibition of LOX [141,142]. The reciprocal expression pattern of miR-30a (Figure 3f), miR-200b [88], and LOX (Figure 3g) is a hallmark of the inflamed colons of IBD patients.

### 5.4. miRNAs Involved in CSC Function

As mentioned before, EMT influences CSC phenotype that, in turn, affects tumor initiation in IBD patients. Expression pattern of microRNAs in an inducible model of CSC formation in breast epithelial cells [168] is considerably similar to that we previously presented in the inflamed colons of IBD patients [37]. Notably, downregulation of miR-200 family members, miR-107, and miR-145 was characteristic in CSCs, while in non-CSCs elevated level of these microRNAs suppressed the expression of EMT inducers, such as ZEB1 and ZEB2 [168].

## 6. Conclusions and Perspectives

In the life of IBD patients, inactive and active disease phases fluctuate, while the position of inflamed and uninflamed colon regions also alternate along the digestive tract. This associates with the fluctuation of inflammation and the altered expression of EMT regulating genes and miRNAs in the colon. Synchronized downregulation of EMT inhibitory miRNAs is a characteristic hallmark in the inflamed colon regions of IBD patients (Figure 2B), which may promote the activation of EMT. Consequently, EMT gives rise to favorable conditions for tumorigenesis by triggering phenotype plasticity, ECM rearrangement, and invasiveness. Continuous activation of inflammatory pathways and EMT produce a supporting microenvironment to initiate and maintain colitis-associated cancer. Besides, pleiotropic microRNAs have the potential to interconnect components of independent processes that eventuate the flare up of IBD, thereby affecting tumorigenesis.

In the complex regulatory system of miRNAs and their target mRNAs, the number of possible interactions is numerous but not infinite. To better understand the molecular interactions underlying the pathogenesis of IBD or IBD-related CRC, systematic examination of the expression of miRNA-mRNA target pairs is of general interest. This is also supported by the recent successful phase IIa studies of miRNA therapy in humans that are all in favor of miRNAs being potential therapeutic targets in IBD.

## Figures and Tables

**Table 1:** Genes involved in EMT and their miRNA regulators. Molecular relation of regulatory miRNAs and their target mRNAs were validated in different model systems.

Relation to EMT	Target Gene		Experimentally Validated Inhibitory miRNA(s)
epithelial markers	*CDH1*	*E-cadherin*	miR-9 [100], miR-25 [102], miR-92a [103]
mesenchymal markers	*CDH2*	*N-cadherin*	miR-194 [107], miR-199a [109], miR-145 [111]
	*VIM*	*vimentin*	miR-30a [112]
	*FN1*	*fibronectin*	miR-200b [115], miR-200c [114]
transcription factors	*SNAI1*	*snail family transcriptional repressor 1*	miR-34a [118], miR-199a [109], miR-30a [119,120]
	*ZEB1*	*zinc finger E-box binding homeobox 1*	miR-200 family [18,121]
	*ZEB2*	*zinc finger E-box binding homeobox 2*	miR-200 family [18,121], miR-192 [122]
	*TWIST1*	*twist family bHLH transcription factor 1*	miR-145 [123]
matrix metalloproteinases	*MMP2*	*matrix metallopeptidase 2*	miR-29b [124]
	*MMP9*	*matrix metallopeptidase 9*	miR-29b [125]
JAK-STAT pathway	*JAK2*	*Janus kinase 2*	miR-375 [126,127]
NOTCH pathway	*NOTCH1*	*notch receptor 1*	miR-34a [128], miR-30a [129]
	*NOTCH2*	*notch receptor 2*	miR-34a [128], miR-30a [129], miR-107 [130]
WNT pathway	*WNT3A*	*Wnt family member 3A*	miR-491 [131]
TGFβ pathway	*SMAD2*	*SMAD family member 2*	miR-200b [99]
Tyrosin kinase receptor associated signaling	*FGF2*	*fibroblast growth factor 2*	miR-194 [132]
	*FGF7*	*fibroblast growth factor 7*	miR-489 [133]
	*AXL*	*AXL receptor tyrosine kinase*	miR-34a [134,135], miR-199a [135], miR-92b [136]
	*EGR1*	*early growth response 1*	miR-192 [122], miR-181a [137]
hypoxia	*HIF1α*	*hypoxia inducible factor 1*	miR-199a [138,139], miR-107 [140]
	*LOX*	*lysyl oxidase*	miR-200b [141], miR-30a [142]
